# Design and Implementation of an Embedded Data Acquisition System for Vehicle Vertical Dynamics Analysis

**DOI:** 10.3390/s23239491

**Published:** 2023-11-29

**Authors:** Joyce Ingrid Venceslau de Souto, Álvaro Barbosa da Rocha, Raimundo Nonato Calazans Duarte, Eisenhawer de Moura Fernandes

**Affiliations:** Department of Mechanical Engineering, Federal University of Campina Grande, Campina Grande 58429900, Brazilalvarobarbosa2@hotmail.com (Á.B.d.R.); nonato.c.duarte@ufcg.edu.br (R.N.C.D.)

**Keywords:** accelerometer, embedded system, microcontroller, vehicle dynamics

## Abstract

With the expansion of electronics in recent decades, it is notorious to observe that embedded systems are increasingly necessary to improve people’s quality of life and to facilitate the diagnosis of systems in general, ranging from pacemakers to control systems. The increased use of electronic components for technological support, such as telemetry systems, electronic injection, and automotive diagnostic scanners, enhances the perspective of data analysis through an embedded system aimed at vehicular systems. Thus, this work aims to design and implement an embedded data acquisition system for the analysis of vehicle vertical dynamics. The methodology for this study was structured into several stages: mathematical modeling of a motorcycle’s mass-spring-damper system, coding for the Arduino microcontroller, computational data analysis supported by MATLAB software version 9.6, electronic prototyping of the embedded system, implementation on the vehicle, and the analysis of motorcycle vertical dynamics parameters. In addition, a mathematical modeling of the mass-spring-damper system was performed using the state-space method. The system was implemented on the Arduino microcontroller platform, enabling real-time data transfer from a motorcycle. The experimental results have successfully validated the proposed data acquisition system.

## 1. Introduction

Studies on vertical dynamics were developed throughout the 20th century through the application of vibration theory to the operating environment of motor vehicles, especially the interaction between the vehicle and the pavement it travels on [1]. Vehicle movement involves traversing various speeds and different types of terrain, resulting in vibration across a broad spectrum of frequencies within its structures [2]. Information about a vehicle’s movement parameters can be employed in the automotive industry to enhance and optimize suspension parameters, mainly those related to vehicle comfort [3]. For instance, in the field of mobile robotics, having access to movement parameters while a wheeled vehicle is in operation enables the real-time calculation of the wheel-terrain friction coefficient. This information can then be applied in control algorithms to enhance the robot’s mobility on challenging terrain, optimize energy usage, and enhance the robot’s autonomous capabilities [4]. Throughout the stages of automobile development, when selecting and analyzing suspension characteristics, it becomes evident that comprehending the interplay between suspension parameters is vital for optimizing this subsystem’s performance [3].

Embedded hardware, combined with software units, is capable of overseeing the mechanical systems of vehicles such as cars and motorcycles. The data acquisition system assesses the vehicle’s physical conditions, transforming the gathered samples into digital numerical values for computational analysis [5]. However, these approaches in the technical literature are primarily tailored for automotive vehicles [6,7,8]. Limited attention has been given to motorcycle applications in existing studies. In these studies, Arduino microcontroller-based systems frequently focus on directly reading sensor parameters or analyzing the entire system holistically without isolating the analysis of subsystems [9,10]. Moreover, there is a lack of real implementation of the system in the vehicle, which hinders data analysis [5,11,12,13]. This limitation becomes especially significant when taking into account the numerous advantages previously described for a thorough evaluation of vehicle dynamics parameters.

Vehicle comfort can be numerically studied in both time and frequency domains. The time-domain approach accommodates non-linear phenomena in vehicle dynamics, while the frequency-domain approach facilitates the formulation of comfort indices [14]. Conversely, conducting experiments with embedded systems that employ accelerometers to characterize driving behavior can also offer valuable insights into investigations on vehicle comfort [15]. To validate the numerical approach, various motion parameter measurement techniques are employed, and among these, the inertial measurement of acceleration followed by integration for velocity or position stands out [4].

This work aims to design and implement an embedded data acquisition system for analyzing vehicle vertical dynamics by obtaining movement parameters in the time domain. The proposed embedded system comprises an Arduino microcontroller integrated with an accelerometer sensor and a memory card module for recording the vehicle’s acceleration. The validation of this proposed prototype is supported by both simulation and experimental results.

## 2. Materials and Methods

### 2.1. Modeling of the Mass-Spring-Damper System

**Figure 1:**
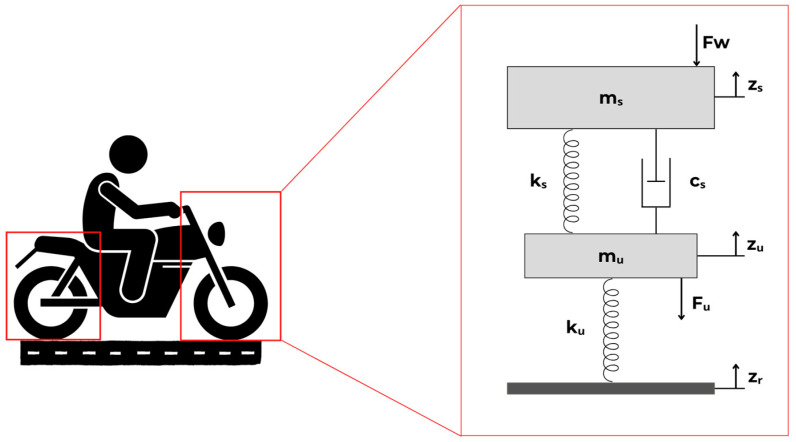
Quarter-car physical model of the motorcycle-rider system.

To analyze a vehicle’s vertical behavior, several ride analysis models were developed, including the quarter-car, half-car, full-vehicle, and multi-body models [2,16]. Among these, the quarter-car model stands out as the simplest to implement due to its straightforward mathematical modeling and quick application [17,18,19]. This model divides the vehicle into sections corresponding to each of the vehicle’s wheels. For motorcycles, the quarter-car model is employed with a single degree of freedom for a mono-suspension (isolated suspension subsystem, either rear or front), with the goal of examining the oscillatory motion concerning road or pavement irregularities (Figure 1).

From Figure 1, *m_u_* indicates the unsprung mass, *m_s_* corresponds to the sprung mass, *k_s_* indicates the suspension spring stiffness, *k_u_* corresponds to the vertical stiffness of the tire, *c_s_* corresponds to the damper damping coefficient, *Z_s_* corresponds to the vertical displacement of the sprung mass, *Z_u_* corresponds to the vertical displacement of the unsprung mass, *Z_R_* corresponds to the vertical displacement imposed by the floor irregularities, *F_b_* indicates the force acting on the sprung mass, and *F_w_* indicates the force acting on the unsprung mass.

The parameters required for conducting comfort analysis using this model are the spring constants and the damping coefficient [20]. Consequently, the damping factor is calculated as (1):

ζ = (c_s_)/(2·(k_s_ · m_s_)^0.5^) (1)

where *ζ* corresponds to the damping factor of the damping system. Conversely, the vertical stiffness of the tire is determined through empirical relations, typically approximated through the Load Deflection relation (LD) [19] as (2):

k_u_ = P_m_/(SLR − UR) (2)

where *k_u_* corresponds to the vertical stiffness of the tire; *P_m_* corresponds to the reference load; *SLR* (Static Loaded Radius) is defined as the distance from the wheel’s center to the ground under reference load and pressure conditions, typically ranging from 15% to 30% of the tire’s section height. *UR* (Unloaded Radius) represents the wheel’s radius without any applied load, also at the reference pressure [16].

Moreover, the State Space representation illustrated in Figure 1 facilitates the simulation of the system’s response to a random input, with the resolution achieved through the Convolution Integral method. This modeling process identifies both the sprung and unsprung masses within the model. The unsprung mass encompasses the suspension systems, wheels, tires, and other components associated with the wheel-tire assembly. Meanwhile, the sprung mass represents the remaining mass of the vehicle [21]. Therefore, the motorcycle is modeled as a system with an ideal mass concentrated at its center of gravity. The methodology employed in this work involves mathematical modeling of the mass-spring-damper system, prototyping the embedded system, and implementing it on the vehicle. The mathematical model of the vehicle was simulated using the MATLAB software version 9.6

### 2.2. Mathematical Modeling of the System

The motorcycle suspension system was modeled through the state space method, which included calculations for both the front and rear suspension configurations. This modeling was based on the quarter-car mathematical model of the vehicle’s mass-spring-damper system (Figure 1). Newton’s Second Law was applied to both the sprung and unsprung masses of the system. To resolve the model, an order reduction was performed in state space form, and the matrix equations of the system, as represented by Equations (3) and (4), were determined; this involved setting the input matrix of the system using the values of *F_w_* and the state vector as the relative displacements between *Z_u_* and *Z_R_* and *Z_s_* and *Z_u_*, along with the velocities of the sprung and unsprung masses.

(3) $$\frac{d}{dt}\left[\begin{array}{c} Z_{u}-Z_{R}\\ \dot{Z_{u}}\\ Z_{s}-Z_{u}\\ \dot{Z_{s}} \end{array}\right]=\left[\begin{array}{cccc} 0 & 1 & 0 & 0\\ \frac{-4k_{s}}{m_{u}} & \frac{-4c_{s}}{m_{u}} & \frac{4k_{u}}{m_{u}} & \frac{4c_{s}}{m_{u}}\\ 0 & -1 & 0 & 1\\ 0 & \frac{4c_{s}}{m_{s}} & \frac{-4k_{s}}{m_{s}} & \frac{-4c_{s}}{m_{s}} \end{array}\right]\left[\begin{array}{c} Z_{u}-Z_{R}\\ \dot{Z_{u}}\\ Z_{s}-Z_{u}\\ \dot{Z_{s}} \end{array}\right]+\left[\begin{array}{c} -1\\ 0\\ 0\\ 0 \end{array}\right]\dot{Z_{R}}$$

(4) $$\left[\begin{array}{c} Y_{1}\\ Y_{2}\\ Y_{3}\\ Y_{4} \end{array}\right]=\left[\begin{array}{cccc} 1 & 0 & 0 & 0\\ 0 & 1 & 0 & 0\\ 0 & 0 & 1 & 0\\ 0 & 0 & 0 & 1 \end{array}\right]\left[\begin{array}{c} Z_{u}-Z_{R}\\ \dot{Z_{u}}\\ Z_{s}-Z_{u}\\ \dot{Z_{s}} \end{array}\right]$$

The mathematical modeling carried out in Equations (3) and (4) was represented in MATLAB from the creation of the system object LTI (linear time-invariant object). The equations *x′* = *Ax* + *Bu* and *y* = *Cx* + *Du* in matrix form (3) and (4) contain matrices A, B, C, and D, which represent the state matrix, the input matrix, the output matrix, and the direct transmission matrix, respectively. The LTI model was solved using the Equations of State method, using the Laplace Transform with the input vector *u*, integrated by the acceleration vector composition. The resolution of the non-homogeneous linear differential equation (*u* ≠ 0) was written considering the experimental values of the pavement excitation force in the time domain as input values to obtain the velocity and displacement.

In order to determine the parameters necessary to obtain the system’s responses to random inputs (rolling track), the theoretical framework was used in conjunction with the service and user manuals for the motorcycle model used in the experimental tests. These parameters included the sprung and unsprung masses, suspension spring stiffness, damping coefficient of the damper, and tire vertical stiffness. For this work, a damping factor of 0.354 was adopted. All values for these parameters are listed in Table 1.

### 2.3. Embedded Data Acquisition System

The embedded system for data acquisition of the motorcycle’s front and rear suspension subsystems was implemented in the Arduino microcontroller Pro Mini version. This choice was driven by its cost-effectiveness and rapid implementation of data acquisition and control algorithms. The Arduino microcontroller is equipped with analog and digital input ports, analog and digital outputs, a communication protocol for programming, and serial communication and operates on a supply voltage of 3.3 and 5.0 V [6]. The transducer employed consists of the MPU-6050 accelerometer and gyroscope, each with 3 axes, resulting in a total of 6 degrees of freedom. The transducer calibration was performed using flat surfaces in the laboratory as a reference to nullify its output. In this work, a first-order digital filter was implemented to ensure a constant sampling rate during readings. Table 2 lists the technical specifications for the MPU-6050 accelerometer.

**Figure 2:**
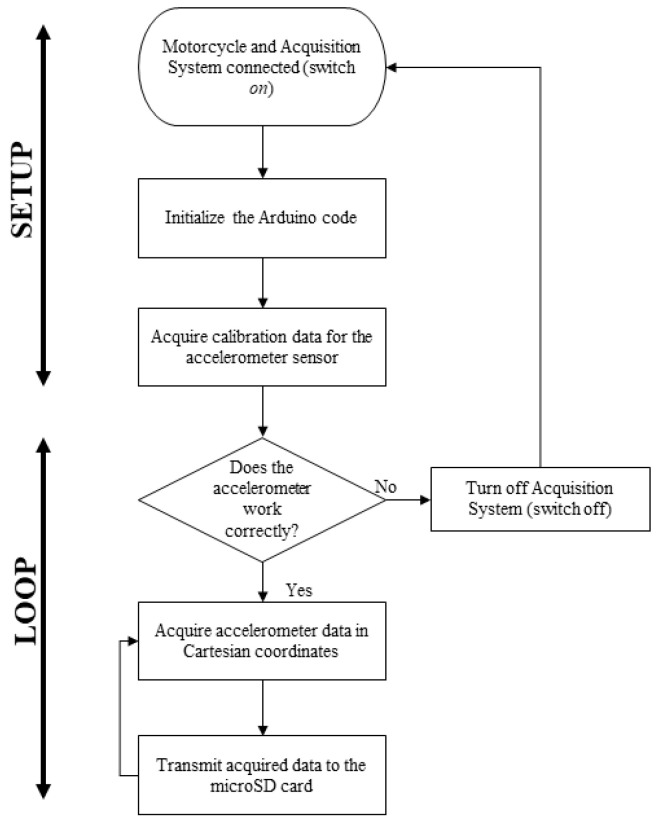
Flowchart of the proposed data acquisition system.

The data collection was stored on a microSD card module, generating a text file with a size of 70 kB, which contained the information recorded during the tests given the sampling period of 0.5 s. In addition, an 18,650 lithium battery (3.7 V) was used to power the acquisition system. Tests were carried out to determine its duration for powering the system considering continuous operation, which was approximately 3 h. The software developed for the system is highly adaptable to meet the requirements of this proposal. The code was written using the Arduino IDE (Integrated Development Environment) environment. Figure 2 shows the flowchart of the implemented program.

**Figure 3:**
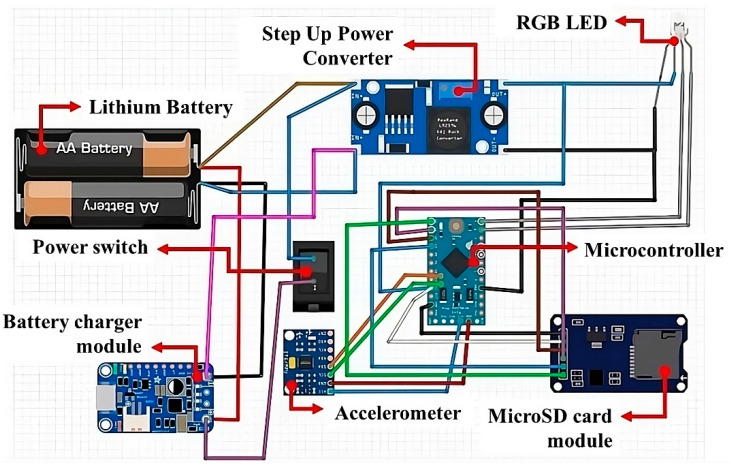
Electronic circuit of the embedded data acquisition system.

The schematic diagram of the proposed data acquisition system based on the Arduino microcontroller is shown in Figure 3.

**Figure 4:**
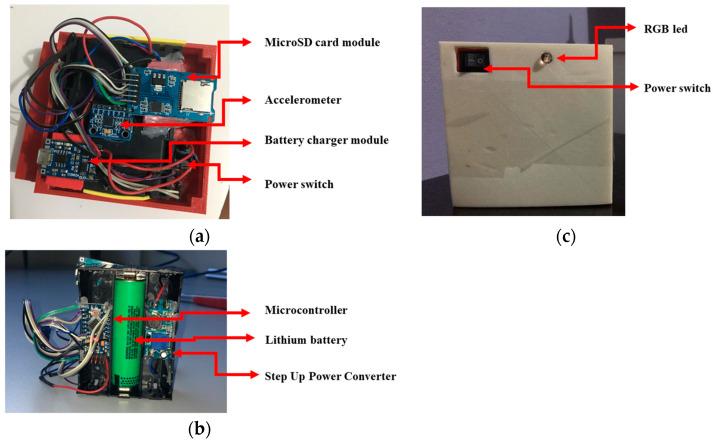
Developed data acquisition system: (**a**) top view, (**b**) bottom view, and (**c**) top view of the ABS enclosure.

The acquisition system was housed in an ABS enclosure developed by FDM additive manufacturing technology. In Figure 4, the developed data acquisition system is presented.

In the construction of the embedded system designed for acquiring suspension data, the project included placing the microcontroller, lithium battery, and step-up converter on one side, while on the opposite side, the accelerometer, microSD card module, battery charger, RGB LED, and power switch were installed. This first stage of building the system made it possible to take the necessary measurements so that the 3D modeling of the case for the DAQ could be carried out in CAD software Autodesk Inventor 2019, specifically Autodesk Inventor ^®^. In the 3D modeling stage, it was decided to model the case as a split piece to facilitate its assembly/disassembly in case of maintenance and component replacement. In addition, as the control of the built system would be performed manually, the user would need to have easy access to the switch and be visually identified when the system was, in fact, operational, that is, ready to acquire data. The materials needed to manufacture and implement the data acquisition system are listed in Table 3. Additionally, the cost of each item is presented.

### 2.4. Vehicle Implementation

**Figure 5:**
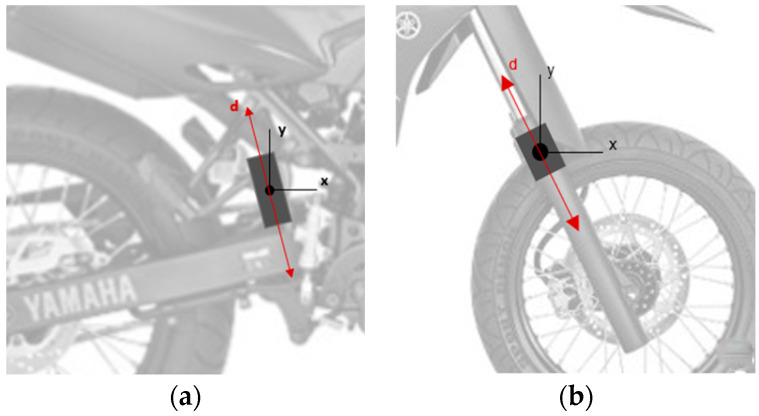
Representation of the installation of the proposed acquisition system in the (**a**) rear and (**b**) front suspension of the motorcycle.

The attachment of the proposed data acquisition modules to the motorcycle was performed as shown in Figure 5, which illustrates the attachment of the system to the motorcycle in the front and rear suspensions of the vehicle. Due to the installation position of the acquisition systems, the vector composition of the Cartesian acceleration components was carried out. According to the fixation of this system, the *x*-axis was adopted as being parallel to the ground.

From the analysis of Figure 5, the vector accelerations of the acquisition system, in the direction d, for the front and rear suspension will be equivalent to the horizontal component x of the acceleration of the embedded system, respectively, in the front and rear suspension.

## 3. Results and Discussions

**Figure 6:**
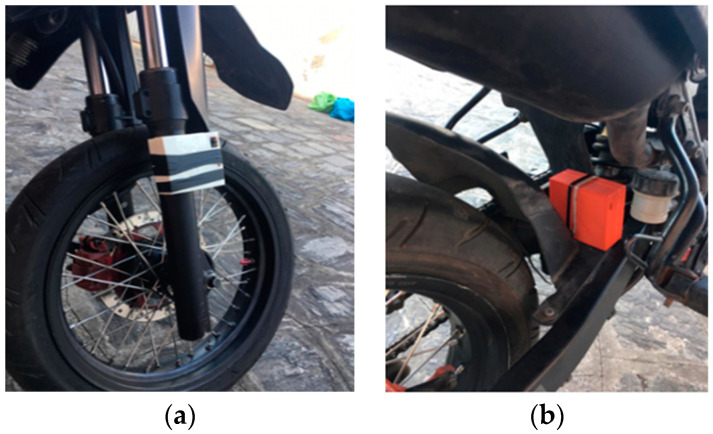
Proposed acquisition systems installed on a motorcycle in the respective subsystems of (**a**) front suspension and (**b**) rear suspension.

After making and installing the acquisition systems in the vehicle (Figure 6), experimental tests were started for predetermined track conditions. In order to analyze the dynamic behavior of the system, two-track conditions were selected that contemplate different types of urban terrain [1,2,19]:

- Condition 1 (C1): cobblestone pavement (170 m);
- Condition 2 (C2): asphalt pavement with three-speed reducers (750 m).

**Figure 7:**
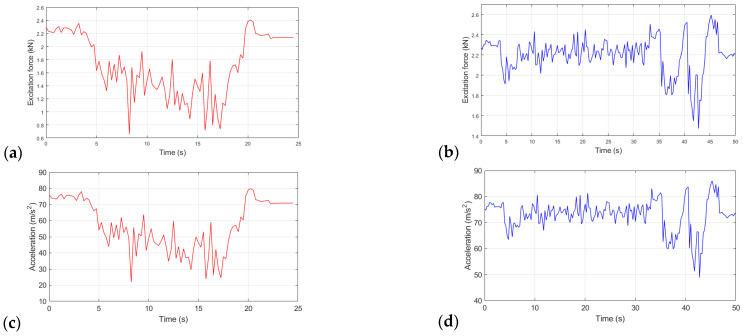
Experimental result of the excitation force on the front suspension in (**a**) C1 and (**b**) C2, in addition to its acceleration in (**c**) C1 and (**d**) C2.

The experimental results consist of the excitation force of the pavement (product of the unsprung mass with the acceleration of the system) in the temporal domain and the velocity and displacement curves of the front and rear system obtained from the elaborated mathematical model. Figure 7 presents the experimental results for the excitation force and acceleration for the front suspension set in test conditions 1 and 2. Specifically, Figure 7a shows the profile of the excitation force measured experimentally by the front suspension in test condition 1. The profile of this magnitude was characterized by intensity variation during the test, ranging from 0.65 kN to 2.4 kN. Figure 7b shows the profile of the excitation force measured by the data acquisition system installed on the front suspension in test condition 2.

For this condition, the behavior of the excitation force presents a distinct variation from the previous situation, showing a short interval of values along the asphalt stretch to the time in its most critical oscillations occurred in the stretches with speed reducers, assuming values in the range of 1.5 kN and 2.6 kN.

**Figure 8:**
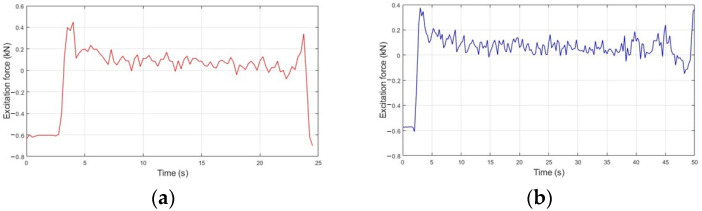

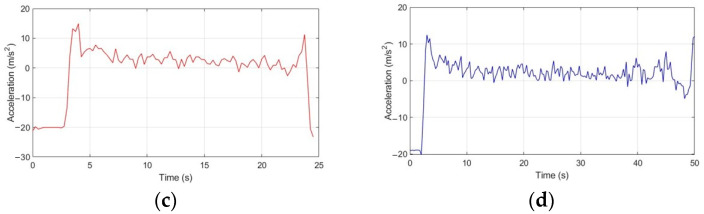
Experimental result of the excitation force on the rear suspension in (**a**) C1 and (**b**) C2, in addition to its acceleration in (**c**) C1 and (**d**) C2.

In Figure 7c, the acceleration curve of the unsprung mass is visualized and obtained by the proposed data acquisition system, referring to the front suspension during the test for condition 1. The acceleration curve for the unsprung mass of the suspension subsystem front travels between the values 22 m/s^2^ and 80 m/s^2^. Furthermore, in Figure 7d, the system’s acceleration curve is produced for the front suspension through C2. Compared to the result of the acceleration curve for condition 1, due to the alpha nature of the track, there is less variability in the assumed values. However, during the passage of the vehicle through the speed reducers, the magnitude travels in the range of 49 m/s^2^ to 85 m/s^2^. Analogously, Figure 8 presents the experimental results for the excitation force and acceleration for the rear suspension set for the two test conditions.

Regarding its analysis, Figure 8a presents the excitation profile of the rear suspension for C1, whose behavior was characterized by variability for an interval of −0.6 kN and 0.45 kN. In Figure 8b, we have the excitation force profile obtained by the system installed in the rear suspension for C2. There is a lower level of variability throughout the test when compared to Figure 8a. Furthermore, the excitation force ranges from −0.58 kN to 0.38 kN. As for Figure 8c, there is the acceleration curve of the unsprung mass for the rear suspension for C1. The magnitude has a profile analogous to the excitation force, presenting values in the range of −20 m/s^2^ to 15 m/s^2^. Additionally, Figure 8d shows the acceleration curve of the system installed in the rear suspension for C2. It is verified that during the passage of the vehicle through the speed reducers, the magnitude travels in the range of −20 m/s^2^ to 12 m/s^2^.

The acceleration value plateau resulting from the excitation force aligns with what has been observed when using the MPU-6050 accelerometer within a data acquisition system designed for a vehicle dynamics application [22]. This observation was made in a simulation environment with predefined excitation frequencies. These acceleration values and their behavior in different track conditions are also observable when employing embedded data acquisition systems utilizing Raspberry Pi and accelerometer-based sensing readings [23]. These experiments were conducted on a bicycle, following a track profile similar to C2.

From the analysis of the experimental results presented in Figure 7 and Figure 8, it can be seen that both suspensions present a very broad behavior regarding the variability of the excitation force throughout the tests, which is more pronounced for C1 due to the inherent characterization of the pavement composed of parallelepiped, also called Belgian Pavé [1]. In relation to C2, the regions with the greatest variation in acceleration, and consequently, in the excitation force, correspond to the instants immediately and after passing through the speed reducers, which are characterized by the deceleration and acceleration of the system.

**Figure 9:**
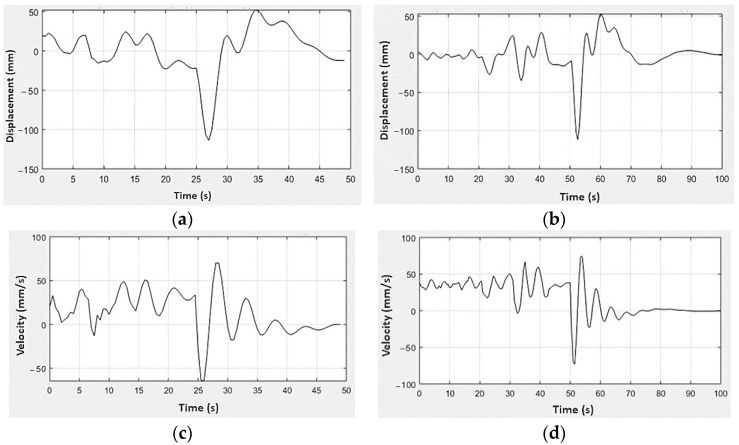
Experimental results of the front suspension for the displacement responses in (**a**) C1 and (**b**) C2, and velocity in (**c**) C1 and (**d**) C2.

**Figure 10:**
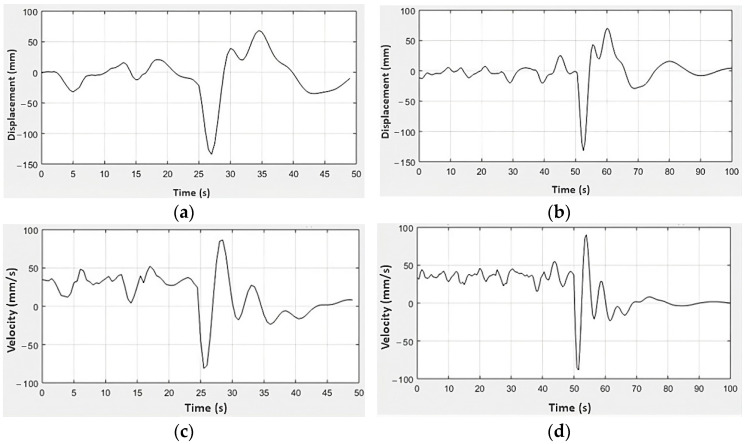
Experimental results of the rear suspension for the displacement responses in (**a**) C1 and (**b**) C2 and velocity in (**c**) C1 and (**d**) C2.

Regarding the acceleration and excitation force profiles of the rear suspension subsystem, the vertical stiffness of the front tire is slightly higher than the rear tire, causing a restriction in the displacement of this subsystem [21], and the natural and working frequency of the rear suspension is higher than the front [18,24]. Compared to the front set, higher absolute values of acceleration and excitation force were observed in the rear suspension set. This characteristic is associated with the real geometric center of the rider-motorcycle set being more directed towards the rear of the motorcycle for this model [25,26] and the spring stiffness constant of the rear suspension being higher than the front [2]. From the experimental results of excitation force and the mathematical model of the system (3)–(4), waveforms of the displacement and velocity signals were found for the front and rear suspension systems of the vehicle (Figure 9 and Figure 10).

Figure 9a presents the results for the front suspension displacement for C1, whose behavior was characterized by a range from −110 mm to 50 mm, similar to Figure 9b results that present the same displacement range for the front suspension through the C2. At the same time, this mechanism is repeated for the rear suspension, whose results are in Figure 10a,b, and which show the same displacement range for different road conditions, from −130 mm to approximately 65 mm. As the reference values for displacement and velocity are taken as the initial resting position of the suspension system, it is understood that positive values refer to the rebound phase of the suspension, while negative values refer to the compression phase of the suspension system. The limits of movement of the front and rear suspensions are intrinsically related to their constructive aspects so as not to exceed their maximum return and compression strokes.

On the other hand, the range of displacement values generated by the excitation force corresponds to the observations made with an Arduino-based data acquisition system applied in a vehicle dynamics context [27]. This system was utilized to assess the responses to diverse track profiles. In general, similarly to Figure 9 and Figure 10, it can be seen that both suspensions have similar displacement ratios throughout test conditions 1 and 2 [21], in addition to greater velocity intervals being reflected in greater transmission of vibrations to the pilot [18,25]. These conditions, together with those already mentioned above, guarantee the comfort and drivability of the vehicle [21].

In this context, the importance of embedded DAQ systems for the analysis of vehicle dynamics is perceived, especially due to the comprehensive ability to customize to specific projects. In the case of automotive dynamics, ADAQ (Arduino Data Acquisition Systems) allows data acquisition with several accelerometers installed in different parts of the vehicle at the same time. In addition, ADAQ is a specific acquisition platform that enables its installation on the vehicle during the time that the investigation lasts without having to mount and dismantle, which is important when you have a considerable number of connected sensors [22]. Additionally, using accelerometers to extract desired information is generally cost-effective, easy to install, and offers relatively low noise levels, making them suitable for a wide range of engineering applications. Unlike displacement sensors, accelerometers do not necessitate fixed reference points, enhancing their convenience in situations where they are often unavailable [28].

## 4. Conclusions

This work presents a microcontroller-based data acquisition system for measuring mechanical acceleration signals and excitation force for motorcycles. The proposed system enables the experimental evaluation of motorcycle velocity and displacement profiles for typical urban routes. The urban test routes consisted of parallelepiped and asphalt pavement with a speed reducer. It was observed that the proposed data acquisition system demonstrated adequate spatial arrangement so that the acceleration and excitation force profiles of the paths were measured coherently with the Cartesian orientation of the subsystems.

With the analysis of the data, the vertical behavior of the motorcycle was obtained as a function of the test condition and the addressed subsystem. Moreover, the developed system underwent experimental validation, affirming its status as a cost-effective solution, with a budget of under USD 40.00, for collecting data on the vehicle’s suspension axles. Its performance aligns with the findings in the existing literature dedicated to embedded systems and data acquisition systems based on Arduino, utilizing the MPU-6050 accelerometer. In addition, the proposed solution is portable and quick to install in the motor vehicle and can be applied to other motor vehicles and other test scenarios for urban and off-road routes.

Based on the results achieved through the implementation of this embedded system, there is a plan to broaden the scope of data processing and human–machine interaction. This expansion will involve the utilization of alternative memory types that can facilitate data acquisition at higher sampling rates. In turn, this will facilitate discussions stemming from the analysis of the frequency spectrum of the data. Additionally, it will allow for data acquisition on extended track routes without being constrained by fixed storage limitations, as is the case with microSD cards. Furthermore, there are plans to enhance the human–machine interface to enable remote and real-time data monitoring, possibly through wireless sensing. Such an approach would eliminate the need for local data storage and open up possibilities for expanding data processing capabilities. Future work will enable a multimodal approach for this data acquisition system project. It is recognized that integrating various sensors into the system, such as temperature sensors, can provide a broader perspective for analyzing factors related to this study. This extension will diversify the discussion of results, including the ability to assess the long-term degradation of shock absorber fluid viscosity and its effects on the motorcycle’s vertical dynamics.

## Figures and Tables

**Table 1:** Parameters of the state space matrixes.

Parameter	Front Suspension	Rear Suspension
m_u_ (kg)	30.15	30.15
m_s_ (kg)	70.35	70.35
k_s_ (N/mm)	4.22	80.00
c_s_ (N·s/mm)	31.53	194.07
k_u_ (N/mm)	115.54	106.19

**Table 2:** MPU-6050 accelerometer technical specifications.

Parameter	Value/Range
Operating voltage (V)	3 to 5
ADC resolution (bits)	16
Gyro range (°/s)	±250, 500, 1000, 2000
Accelerometer range (g; 9.81 m/s^2^)	±2, ±4, ±8, ±16
Temperature sensor reading (°C)	40 to 85

**Table 3:** The set of components and their respective cost for the data acquisition system design.

Component	Quantity	Unitary Value ($)
Arduino Pro Mini	2	5.04
Accelerometer MPU-6050	2	3.36
MicroSD card module	2	2.61
TP4056 lithium battery charger module	2	1.29
MT3608 Step-Up Boost Power Converter	2	2.02
Power rocker switch	2	0.36
RGB Led	2	0.12
18650 Battery holder 3 Slots	2	1.97
18650 Lithium battery	2	3.17
	Total	39.88

## Data Availability

The data presented in this study are available on request from the corresponding author.
